# 3-Ethyl­sulfinyl-2-(4-fluoro­phen­yl)-5-iodo-1-benzofuran

**DOI:** 10.1107/S1600536810012717

**Published:** 2010-04-10

**Authors:** Hong Dae Choi, Pil Ja Seo, Byeng Wha Son, Uk Lee

**Affiliations:** a Department of Chemistry, Dongeui University, San 24 Kaya-dong Busanjin-gu, Busan 614-714, Republic of Korea; b Department of Chemistry, Pukyong National University, 599-1 Daeyeon 3-dong, Nam-gu, Busan 608-737, Republic of Korea

## Abstract

In the title compound, C_16_H_12_FIO_2_S, the 4-fluoro­phenyl ring is rotated slightly out of the benzofuran plane, as indicated by the dihedral angle of 4.48 (5)°. In the crystal structure, pairs of I⋯O halogen bonds [I⋯O = 3.123 (1) Å] link the mol­ecules into centrosymmetric dimers. These dimers are further linked *via* aromatic π–π inter­actions between the benzene and 4-fluoro­phenyl rings of neighbouring mol­ecules [centroid–centroid distance = 3.620 (3) Å].

## Related literature

For the crystal structures of similar 3-ethyl­sulfinyl-2-(4-fluoro­phen­yl)-5-halo-1-benzofuran derivatives, see: Choi *et al.* (2010**a* ▶,*b* ▶,c* ▶). For the pharmacological activity of benzofuran compounds, see: Aslam *et al.* (2006 ▶); Galal *et al.* (2009 ▶); Khan *et al.* (2005 ▶). For natural products with benzofuran rings, see: Akgul & Anil (2003 ▶); Soekamto *et al.* (2003 ▶). For a review of halogen bonding, see: Politzer *et al.* (2007 ▶).
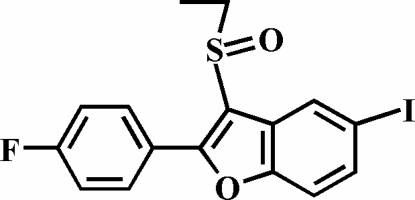


## Experimental

### 

#### Crystal data

C_16_H_12_FIO_2_S
*M* *_r_* = 414.22
Triclinic, 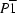

*a* = 7.2942 (4) Å
*b* = 9.6312 (5) Å
*c* = 11.0132 (6) Å
α = 100.637 (2)°
β = 97.947 (2)°
γ = 105.086 (2)°
*V* = 719.91 (7) Å^3^
*Z* = 2
Mo *K*α radiation
μ = 2.38 mm^−1^
*T* = 173 K
0.38 × 0.18 × 0.11 mm

#### Data collection

Bruker SMART APEXII CCD diffractometer
Absorption correction: multi-scan (*SADABS*; Bruker, 2009 ▶) *T*_min_ = 0.548, *T*_max_ = 0.746
12738 measured reflections
3342 independent reflections
3237 reflections with *I* > 2σ(*I*)
*R*_int_ = 0.029

#### Refinement

*R*[*F* ^2^ > 2σ(*F* ^2^)] = 0.019
*wR*(*F* ^2^) = 0.052
*S* = 1.11
3342 reflections
191 parameters
H-atom parameters constrained
Δρ_max_ = 0.37 e Å^−3^
Δρ_min_ = −0.63 e Å^−3^

### 

Data collection: *APEX2* (Bruker, 2009 ▶); cell refinement: *SAINT* (Bruker, 2009 ▶); data reduction: *SAINT*; program(s) used to solve structure: *SHELXS97* (Sheldrick, 2008 ▶); program(s) used to refine structure: *SHELXL97* (Sheldrick, 2008 ▶); molecular graphics: *ORTEP-3* (Farrugia, 1997 ▶) and *DIAMOND* (Brandenburg, 1998 ▶); software used to prepare material for publication: *SHELXL97*.

## Supplementary Material

Crystal structure: contains datablocks global, I. DOI: 10.1107/S1600536810012717/ds2026sup1.cif

Structure factors: contains datablocks I. DOI: 10.1107/S1600536810012717/ds2026Isup2.hkl

Additional supplementary materials:  crystallographic information; 3D view; checkCIF report

## Figures and Tables

**Fig. 1.**
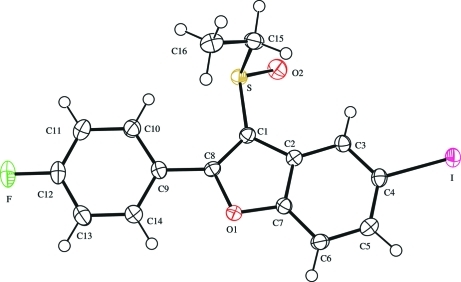
The molecular structure of the title compound with the atom numbering scheme. Displacement ellipsoids are drawn at the 50% probability level. H atoms are presented as a small spheres of arbitrary radius.

**Fig. 2.**
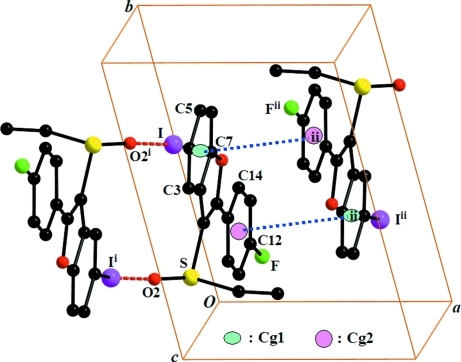
I···O and π–π interactions (dotted lines) in the crystal structure of the title compound. Cg denotes the ring centroids. [Symmetry codes: (i) - x, - y + 1, - z + 2; (ii) - x + 1, - y + 1, - z + 1.]

## supplementary crystallographic information

### Comment

Compounds containing benzofuran moiety show interesting pharmacological activities such as antifungal (Aslam *et al.*, 2006), antitumor and antiviral (Galal *et al.*, 2009), antimicrobial (Khan *et al*., 2005) properties. These compounds are widely occurring in nature (Akgul & Anil, 2003; Soekamto *et al.*, 2003). As a part of our ongoing studies of the effect of side chain substituents on the solid state structures of 3-ethylsulfinyl-2-(4-fluorophenyl)-5-halo-1-benzofuran analogues (Choi *et al.*, 2010*a,b,c*), we report the crystal structure of the title compound (Fig. 1).

The benzofuran unit is essentially planar, with a mean deviation of 0.009 (1) Å from the least-squares plane defined by the nine constituent atoms. The dihedral angle formed by the benzofuran plane and the 4-fluorophenyl ring is 4.48 (5)°. The crystal packing (Fig. 2) is stabilized by I···O halogen bondings between the iodine and the oxygen of the S═O unit [I···O2^i^ = 3.123 (1) Å; C–I···O2^i^ = 167.88 (5)°] (Politzer *et al.*,  2007). The molecular packing (Fig. 2) is further stabilized by aromatic π–π interactions between the benzene and the 4-fluorophenyl rings of adjacent molecules, with a Cg1···Cg2^ii^ distance of 3.620 (3) Å (Cg1 and Cg2 are the centroids of the the C2-C7 benzene ring and the C9-C14 4-fluorophenyl ring, respectively).

### Experimental

77% 3-Chloroperoxybenzoic acid (202 mg, 0.9 mmol) was added in small portions to a stirred solution of 3-ethylsulfanyl-2-(4-fluorophenyl)-5-iodo-1-benzofuran (358 mg, 0.9 mmol) in dichloromethane (30 mL) at 273 K. After being stirred at room temperature for 4h, the mixture was washed with saturated sodium bicarbonate solution and the organic layer was separated, dried over magnesium sulfate, filtered and concentrated at reduced pressure. The residue was purified by column chromatography (hexane-ethyl acetate, 1:1 v/v) to afford the title compound as a colorless solid [yield 80%, m.p. 446-447 K; *R*_f_ = 0.51 (hexane-ethyl acetate, 1:1 v/v)]. Single crystals suitable for X-ray diffraction were prepared by slow evaporation of a solution of the title compound in chloroform at room temperature.

### Refinement

All H atoms were positioned geometrically and refined using a riding model, with C-H = 0.95 Å for aryl, 0.98 Å for methylene, and 0.00 Å for methyl H atoms. *U*_iso_(H)= 1.2*U*_eq_(C) for aryl and methylene H atoms, and 1.5*U*_eq_(C) for methyl H atoms.

### Crystal data

**Table:**

C_16_H_12_FIO_2_S	*Z* = 2
*M**_r_* = 414.22	*F*(000) = 404
Triclinic, *P*1	*D*_x_ = 1.911 Mg m^−^^3^
Hall symbol: -P 1	Mo *K*α radiation, λ = 0.71073 Å
*a* = 7.2942 (4) Å	Cell parameters from 9908 reflections
*b* = 9.6312 (5) Å	θ = 2.6–27.6°
*c* = 11.0132 (6) Å	µ = 2.38 mm^−^^1^
α = 100.637 (2)°	*T* = 173 K
β = 97.947 (2)°	Block, colourless
γ = 105.086 (2)°	0.38 × 0.18 × 0.11 mm
*V* = 719.91 (7) Å^3^	

### Data collection

**Table:**

Bruker SMART APEXII CCD diffractometer	3342 independent reflections
Radiation source: Rotating Anode	3237 reflections with *I* > 2σ(*I*)
Bruker HELIOS graded multilayer optics	*R*_int_ = 0.029
Detector resolution: 10.0 pixels mm^-1^	θ_max_ = 27.6°, θ_min_ = 1.9°
φ and ω scans	*h* = −9→9
Absorption correction: multi-scan (*SADABS*; Bruker, 2009)	*k* = −12→12
*T*_min_ = 0.548, *T*_max_ = 0.746	*l* = −14→14
12738 measured reflections	

### Refinement

**Table:**

Refinement on *F*^2^	Primary atom site location: structure-invariant direct methods
Least-squares matrix: full	Secondary atom site location: difference Fourier map
*R*[*F*^2^ > 2σ(*F*^2^)] = 0.019	Hydrogen site location: difference Fourier map
*wR*(*F*^2^) = 0.052	H-atom parameters constrained
*S* = 1.11	*w* = 1/[σ^2^(*F*_o_^2^) + (0.0269*P*)^2^ + 0.2687*P*] where *P* = (*F*_o_^2^ + 2*F*_c_^2^)/3
3342 reflections	(Δ/σ)_max_ < 0.001
191 parameters	Δρ_max_ = 0.37 e Å^−^^3^
0 restraints	Δρ_min_ = −0.63 e Å^−^^3^

### Special details

**Table:**

Geometry. All esds (except the esd in the dihedral angle between two l.s. planes) are estimated using the full covariance matrix. The cell esds are taken into account individually in the estimation of esds in distances, angles and torsion angles; correlations between esds in cell parameters are only used when they are defined by crystal symmetry. An approximate (isotropic) treatment of cell esds is used for estimating esds involving l.s. planes.
Refinement. Refinement of F^2^ against ALL reflections. The weighted R-factor wR and goodness of fit S are based on F^2^, conventional R-factors R are based on F, with F set to zero for negative F^2^. The threshold expression of F^2^ > 2sigma(F^2^) is used only for calculating R-factors(gt) etc. and is not relevant to the choice of reflections for refinement. R-factors based on F^2^ are statistically about twice as large as those based on F, and R- factors based on ALL data will be even larger.

### Fractional atomic coordinates and isotropic or equivalent isotropic displacement parameters (Å^2^)

**Table:**

	*x*	*y*	*z*	*U*_iso_*/*U*_eq_	
I	0.226555 (16)	0.736460 (12)	1.062556 (10)	0.02602 (5)	
S	0.04077 (6)	0.19740 (5)	0.59260 (4)	0.02146 (9)	
F	0.23547 (19)	0.15061 (15)	−0.01197 (11)	0.0389 (3)	
O1	0.29054 (17)	0.57530 (13)	0.50405 (11)	0.0205 (2)	
O2	−0.0999 (2)	0.20821 (16)	0.67760 (14)	0.0322 (3)	
C1	0.1561 (2)	0.37895 (18)	0.57995 (16)	0.0197 (3)	
C2	0.2016 (2)	0.51016 (18)	0.68023 (16)	0.0190 (3)	
C3	0.1835 (2)	0.53995 (19)	0.80655 (16)	0.0207 (3)	
H3	0.1287	0.4630	0.8455	0.025*	
C4	0.2487 (2)	0.68639 (19)	0.87246 (16)	0.0213 (3)	
C5	0.3272 (3)	0.8028 (2)	0.81686 (18)	0.0246 (4)	
H5	0.3683	0.9018	0.8652	0.030*	
C6	0.3448 (3)	0.77340 (19)	0.69133 (17)	0.0231 (3)	
H6	0.3972	0.8502	0.6517	0.028*	
C7	0.2821 (2)	0.62657 (18)	0.62715 (16)	0.0193 (3)	
C8	0.2123 (2)	0.42343 (18)	0.47623 (16)	0.0189 (3)	
C9	0.2136 (2)	0.35025 (19)	0.34774 (16)	0.0199 (3)	
C10	0.1546 (3)	0.1962 (2)	0.30668 (18)	0.0255 (4)	
H10	0.1113	0.1371	0.3632	0.031*	
C11	0.1583 (3)	0.1286 (2)	0.18520 (19)	0.0290 (4)	
H11	0.1152	0.0240	0.1571	0.035*	
C12	0.2255 (3)	0.2160 (2)	0.10629 (17)	0.0269 (4)	
C13	0.2847 (3)	0.3683 (2)	0.14173 (18)	0.0282 (4)	
H13	0.3299	0.4258	0.0846	0.034*	
C14	0.2767 (3)	0.4354 (2)	0.26295 (17)	0.0252 (3)	
H14	0.3144	0.5402	0.2887	0.030*	
C15	0.2486 (3)	0.1670 (2)	0.68306 (18)	0.0267 (4)	
H15A	0.3105	0.2532	0.7547	0.032*	
H15B	0.2047	0.0789	0.7178	0.032*	
C16	0.3954 (3)	0.1442 (2)	0.6025 (2)	0.0296 (4)	
H16A	0.3359	0.0565	0.5335	0.044*	
H16B	0.5069	0.1304	0.6543	0.044*	
H16C	0.4384	0.2310	0.5676	0.044*	

### Atomic displacement parameters (Å^2^)

**Table:**

	*U*^11^	*U*^22^	*U*^33^	*U*^12^	*U*^13^	*U*^23^
I	0.03240 (8)	0.02619 (8)	0.01896 (8)	0.01017 (5)	0.00499 (5)	0.00175 (5)
S	0.02080 (19)	0.01889 (19)	0.0227 (2)	0.00112 (15)	0.00640 (15)	0.00498 (15)
F	0.0464 (7)	0.0480 (7)	0.0218 (6)	0.0179 (6)	0.0109 (5)	−0.0020 (5)
O1	0.0239 (6)	0.0181 (6)	0.0198 (6)	0.0050 (5)	0.0068 (4)	0.0052 (4)
O2	0.0285 (7)	0.0332 (7)	0.0361 (8)	0.0043 (6)	0.0180 (6)	0.0092 (6)
C1	0.0184 (7)	0.0185 (8)	0.0214 (8)	0.0039 (6)	0.0045 (6)	0.0039 (6)
C2	0.0166 (7)	0.0191 (7)	0.0213 (8)	0.0052 (6)	0.0045 (6)	0.0043 (6)
C3	0.0193 (7)	0.0226 (8)	0.0208 (8)	0.0057 (6)	0.0060 (6)	0.0051 (6)
C4	0.0202 (7)	0.0247 (8)	0.0197 (8)	0.0090 (6)	0.0045 (6)	0.0027 (6)
C5	0.0254 (8)	0.0193 (8)	0.0279 (9)	0.0069 (7)	0.0041 (7)	0.0027 (7)
C6	0.0254 (8)	0.0186 (8)	0.0261 (9)	0.0062 (6)	0.0068 (7)	0.0065 (6)
C7	0.0189 (7)	0.0205 (8)	0.0200 (8)	0.0072 (6)	0.0052 (6)	0.0050 (6)
C8	0.0172 (7)	0.0168 (7)	0.0222 (8)	0.0046 (6)	0.0039 (6)	0.0040 (6)
C9	0.0171 (7)	0.0246 (8)	0.0190 (8)	0.0076 (6)	0.0039 (6)	0.0048 (6)
C10	0.0271 (9)	0.0231 (8)	0.0258 (9)	0.0056 (7)	0.0084 (7)	0.0046 (7)
C11	0.0297 (9)	0.0254 (9)	0.0284 (9)	0.0077 (7)	0.0053 (7)	−0.0013 (7)
C12	0.0251 (8)	0.0363 (10)	0.0189 (8)	0.0131 (8)	0.0040 (6)	0.0000 (7)
C13	0.0304 (9)	0.0360 (10)	0.0222 (9)	0.0126 (8)	0.0079 (7)	0.0102 (8)
C14	0.0277 (9)	0.0251 (9)	0.0236 (9)	0.0082 (7)	0.0055 (7)	0.0061 (7)
C15	0.0295 (9)	0.0243 (9)	0.0266 (9)	0.0068 (7)	0.0040 (7)	0.0096 (7)
C16	0.0249 (8)	0.0261 (9)	0.0375 (11)	0.0073 (7)	0.0039 (8)	0.0085 (8)

### Geometric parameters (Å, °)

**Table:**

I—C4	2.098 (2)	C6—H6	0.9500
I—O2^i^	3.123 (1)	C8—C9	1.462 (2)
S—O2	1.492 (1)	C9—C10	1.399 (2)
S—C1	1.772 (2)	C9—C14	1.401 (2)
S—C15	1.816 (2)	C10—C11	1.383 (3)
F—C12	1.358 (2)	C10—H10	0.9500
O1—C7	1.370 (2)	C11—C12	1.370 (3)
O1—C8	1.382 (2)	C11—H11	0.9500
C1—C8	1.369 (2)	C12—C13	1.379 (3)
C1—C2	1.446 (2)	C13—C14	1.388 (3)
C2—C7	1.392 (2)	C13—H13	0.9500
C2—C3	1.399 (2)	C14—H14	0.9500
C3—C4	1.386 (2)	C15—C16	1.515 (3)
C3—H3	0.9500	C15—H15A	0.9900
C4—C5	1.406 (3)	C15—H15B	0.9900
C5—C6	1.390 (3)	C16—H16A	0.9800
C5—H5	0.9500	C16—H16B	0.9800
C6—C7	1.383 (2)	C16—H16C	0.9800
			
C4—I—O2^i^	167.88 (5)	C10—C9—C8	121.74 (16)
O2—S—C1	107.52 (8)	C14—C9—C8	119.67 (15)
O2—S—C15	107.08 (9)	C11—C10—C9	121.07 (17)
C1—S—C15	97.46 (8)	C11—C10—H10	119.5
C7—O1—C8	107.01 (13)	C9—C10—H10	119.5
C8—C1—C2	107.24 (14)	C12—C11—C10	118.44 (17)
C8—C1—S	127.97 (13)	C12—C11—H11	120.8
C2—C1—S	124.75 (13)	C10—C11—H11	120.8
C7—C2—C3	119.36 (16)	F—C12—C11	118.94 (17)
C7—C2—C1	105.00 (15)	F—C12—C13	118.20 (18)
C3—C2—C1	135.64 (16)	C11—C12—C13	122.86 (17)
C4—C3—C2	117.18 (16)	C12—C13—C14	118.37 (18)
C4—C3—H3	121.4	C12—C13—H13	120.8
C2—C3—H3	121.4	C14—C13—H13	120.8
C3—C4—C5	122.70 (16)	C13—C14—C9	120.64 (17)
C3—C4—I	118.59 (13)	C13—C14—H14	119.7
C5—C4—I	118.71 (13)	C9—C14—H14	119.7
C6—C5—C4	120.18 (16)	C16—C15—S	111.38 (13)
C6—C5—H5	119.9	C16—C15—H15A	109.4
C4—C5—H5	119.9	S—C15—H15A	109.4
C7—C6—C5	116.55 (16)	C16—C15—H15B	109.4
C7—C6—H6	121.7	S—C15—H15B	109.4
C5—C6—H6	121.7	H15A—C15—H15B	108.0
O1—C7—C6	125.25 (15)	C15—C16—H16A	109.5
O1—C7—C2	110.72 (14)	C15—C16—H16B	109.5
C6—C7—C2	124.02 (16)	H16A—C16—H16B	109.5
C1—C8—O1	110.02 (14)	C15—C16—H16C	109.5
C1—C8—C9	135.88 (16)	H16A—C16—H16C	109.5
O1—C8—C9	114.08 (14)	H16B—C16—H16C	109.5
C10—C9—C14	118.59 (16)		
			
O2—S—C1—C8	−143.28 (16)	C2—C1—C8—O1	−0.15 (18)
C15—S—C1—C8	106.12 (17)	S—C1—C8—O1	177.70 (12)
O2—S—C1—C2	34.22 (16)	C2—C1—C8—C9	177.97 (17)
C15—S—C1—C2	−76.38 (15)	S—C1—C8—C9	−4.2 (3)
C8—C1—C2—C7	0.56 (18)	C7—O1—C8—C1	−0.33 (18)
S—C1—C2—C7	−177.37 (12)	C7—O1—C8—C9	−178.90 (13)
C8—C1—C2—C3	−179.12 (18)	C1—C8—C9—C10	−3.3 (3)
S—C1—C2—C3	2.9 (3)	O1—C8—C9—C10	174.74 (15)
C7—C2—C3—C4	−0.2 (2)	C1—C8—C9—C14	177.15 (19)
C1—C2—C3—C4	179.41 (18)	O1—C8—C9—C14	−4.8 (2)
C2—C3—C4—C5	1.1 (2)	C14—C9—C10—C11	0.1 (3)
C2—C3—C4—I	−179.54 (12)	C8—C9—C10—C11	−179.44 (16)
C3—C4—C5—C6	−1.0 (3)	C9—C10—C11—C12	1.5 (3)
I—C4—C5—C6	179.69 (13)	C10—C11—C12—F	177.94 (16)
C4—C5—C6—C7	−0.1 (3)	C10—C11—C12—C13	−1.8 (3)
C8—O1—C7—C6	−179.49 (16)	F—C12—C13—C14	−179.32 (16)
C8—O1—C7—C2	0.71 (17)	C11—C12—C13—C14	0.4 (3)
C5—C6—C7—O1	−178.78 (15)	C12—C13—C14—C9	1.3 (3)
C5—C6—C7—C2	1.0 (3)	C10—C9—C14—C13	−1.5 (3)
C3—C2—C7—O1	178.96 (14)	C8—C9—C14—C13	178.04 (16)
C1—C2—C7—O1	−0.79 (18)	O2—S—C15—C16	176.25 (13)
C3—C2—C7—C6	−0.8 (2)	C1—S—C15—C16	−72.79 (14)
C1—C2—C7—C6	179.41 (16)		

Symmetry codes: (i) −*x*, −*y*+1, −*z*+2.
